# Modification of Brain Oscillations via Rhythmic Light Stimulation Provides Evidence for Entrainment but Not for Superposition of Event-Related Responses

**DOI:** 10.3389/fnhum.2016.00010

**Published:** 2016-02-03

**Authors:** Annika Notbohm, Jürgen Kurths, Christoph S. Herrmann

**Affiliations:** ^1^Experimental Psychology Lab, Center for Excellence ‘Hearing4all’, European Medical School, University of OldenburgOldenburg, Germany; ^2^Potsdam Institute for Climate Impact ResearchPotsdam, Germany; ^3^Research Center Neurosensory Science, University of OldenburgOldenburg, Germany

**Keywords:** alpha, visual flicker, individual alpha frequency, Arnold tongue, intermittency, parietal cortex, steady-state visually evoked potential (SSVEP), superposition

## Abstract

The functional relevance of brain oscillations in the alpha frequency range (8–13 Hz) has been repeatedly investigated through the use of rhythmic visual stimulation. The underlying mechanism of the steady-state visual evoked potential (SSVEP) measured in EEG during rhythmic stimulation, however, is not known. There are two hypotheses on the origin of SSVEPs: entrainment of brain oscillations and superposition of event-related responses (ERPs). The entrainment but not the superposition hypothesis justifies rhythmic visual stimulation as a means to manipulate brain oscillations, because superposition assumes a linear summation of single responses, independent from ongoing brain oscillations. Here, we stimulated participants with a rhythmic flickering light of different frequencies and intensities. We measured entrainment by comparing the phase coupling of brain oscillations stimulated by rhythmic visual flicker with the oscillations induced by arrhythmic jittered stimulation, varying the time, stimulation frequency, and intensity conditions. In line with a theoretical concept of entrainment (the so called *Arnold tongue*), we found the phase coupling to be more pronounced with increasing stimulation intensity as well as at stimulation frequencies closer to each participant's intrinsic frequency. Only inside the Arnold tongue did the conditions significantly differ from the jittered stimulation. Furthermore, even in a single sequence of an SSVEP, we found non-linear features (intermittency of phase locking) that contradict the linear summation of single responses, as assumed by the superposition hypothesis. Our findings provide unequivocal evidence that visual rhythmic stimulation entrains brain oscillations, thus validating the approach of rhythmic stimulation as a manipulation of brain oscillations.

## Introduction

A relationship between ongoing alpha amplitudes and perception has been a subject of investigation since Berger's findings in the late 1920s (Berger, 1929). He described an occipital alpha-amplitude decline after stimulation, which was later shown to reflect event-related desynchronization of the ongoing alpha rhythm (Pfurtscheller and Aranibar, 1977). Experimental data largely reveal this cortical state to represent an electrophysiological correlate of activation (Arieli et al., 1996; von Stein and Sarnthein, 2000), whereas an increased amplitude in the alpha range co-occurs with inhibited perception (Klimesch et al., 2007). Besides the amplitude, alpha phase angles were found to correlate with behavior (Hanslmayr et al., 2007; Klimesch et al., 2007). These findings support the key role of brain oscillations in the perception process (Hanslmayr et al., 2005; Lakatos et al., 2008; Busch et al., 2009; Jensen and Mazaheri, 2010). It is yet unclear, however, whether oscillations indeed reflect a fundamental mechanism of information processing or rather appear as an epiphenomenon (Buzsáki and Draguhn, 2004; Herrmann et al., 2015).

Numerous studies report a modification of brain oscillations, making considerable gains in addressing this question. Besides transcranial approaches, such as magnetic stimulation (TMS; e.g., Dugué et al., 2011; Romei et al., 2011) or alternating current stimulation (tACS, Zaehle et al., 2010; Helfrich et al., 2014) a rhythmic flickering light can serve as a stimulation source. Behavioral studies show that a stream of rhythmic light stimuli improves the perception of targets presented in phase with the rhythmic stimuli (Mathewson et al., 2012; de Graaf et al., 2013; Spaak et al., 2014). Such rhythmic presentation of visual stimuli results in an electrophysiological signal, referred to as a steady-state visual evoked potential (SSVEP) in humans (Regan, 1982; Herrmann, 2001; Müller et al., 2003; Di Russo et al., 2007) as well as in animals (Mechler et al., 1998; Xu et al., 2013). The SSVEP is believed to derive from entrainment of an internal oscillator by an external visual rhythmic driving force. In the occipital cortex, this internal oscillator oscillates at the individual alpha frequency (IAF), which is then shifted toward the external frequency during entrainment.

Entrainment as the fundamental mechanism of SSVEPs is a basic requirement when applying rhythmic stimulation to investigate the causal link between oscillations and behavior (Thut et al., 2011), as the term *entrainment* implies interference with the ongoing brain oscillations. Recently, however, it has been questioned whether the SSVEP indeed reflects entrainment. While some studies report an interaction of stimulation and internal oscillations (Schwab et al., 2006; Halbleib et al., 2012), others found the stimulation to produce a series of event-related responses (ERPs), superimposing to a nearly sinusoidal oscillatory response (Capilla et al., 2011). Furthermore, Keitel et al. (2014) identified different topographies of SSVEPs and internal oscillations, thus indicating a lack of clear understanding of the underlying mechanism of the SSVEP. The superposition hypothesis entails independence of SSVEPs and the ongoing oscillations (Capilla et al., 2011). Hence, if the superposition of ERPs can indeed fully explain SSVEPs and entrainment cannot be demonstrated, altered behavior as a consequence of rhythmic light stimulation could not be considered evidence of the functional connection between oscillations and behavior.

**Figure 1:**
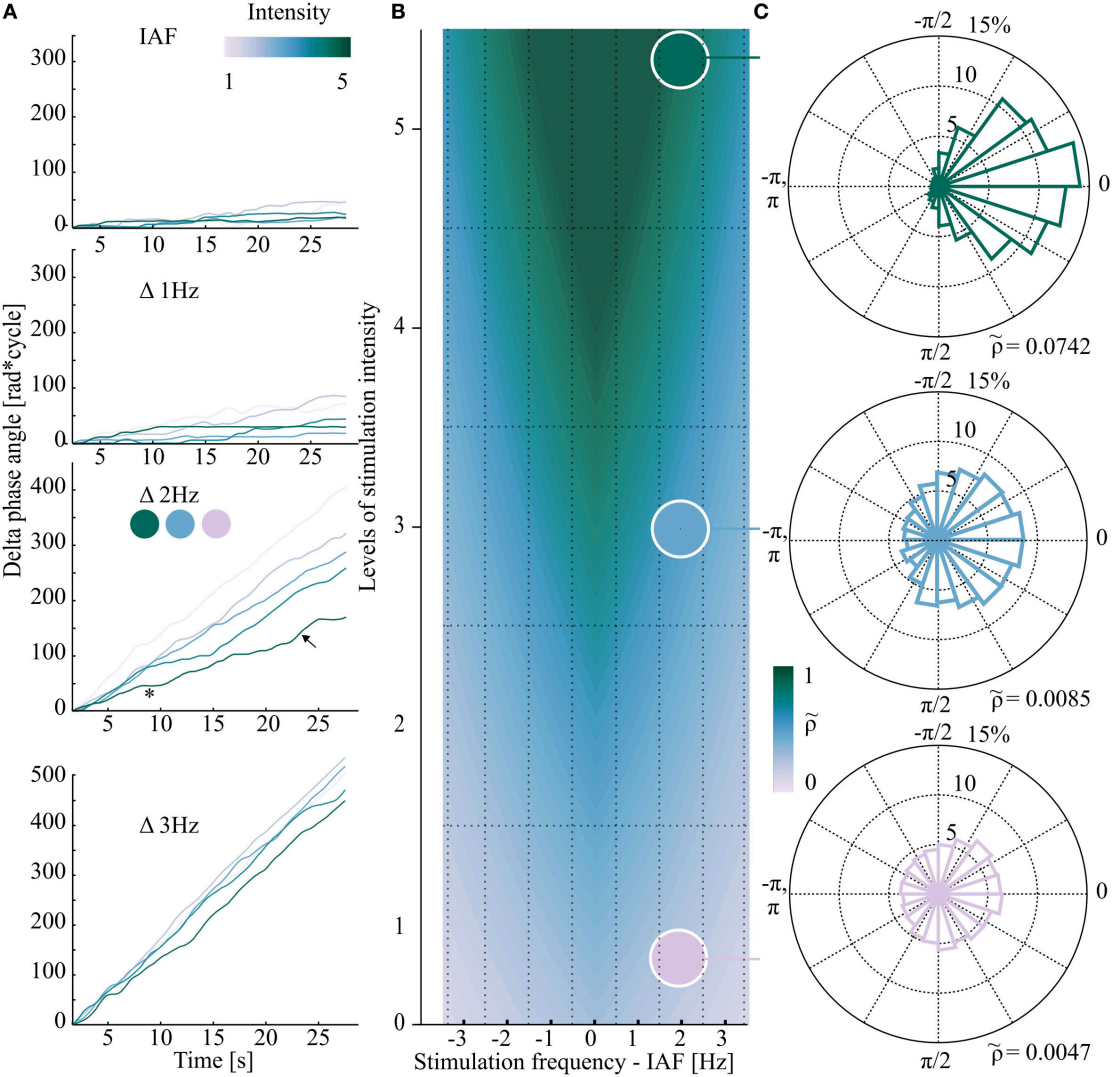
**Exemplary data (one representative subject in A,C) from different areas of the schematic Arnold tongue (synthetic data in B)**. If the stimulation properties (frequency/intensity) fall inside the Arnold tongue (triangular shaped color gradient in **B**), EEG and flicker show phase clustering (green phase plot in **C**) while outside the Arnold tongue Δ phase angles are randomly distributed (lilac phase plot in **C**). **(A)** Phase difference between stimulation and EEG signal over time. Each plot represents one stimulation frequency, from IAF (individual alpha frequency, Δ0 Hz) to Δ3 Hz in 1 Hz steps. All five stimulation intensities are shown. The asterisk and arrow in **(A)** mark an exemplary horizontal plateau of zero phase difference (synchronization, ^*^), and an exemplary phase slip (↖), respectively. At Δ3 Hz phase drift was observed for the five intensities. **(B)** Schematic view of the hypothesized Arnold tongue (synthetic data). Within the Arnold tongue (green area) the intrinsic oscillator (EEG) is synchronized to the external stimulation. Outside the Arnold tongue (lilac area) EEG and external stimulation constantly show a phase drift. At the border (blue area), switches between the two states can be observed. Each square of the grid demonstrates one of the 35 stimulation condition. **(C)** Based on the data from **(A)** (third plot), three exemplary polar plots of the distribution of phase differences are shown for Δ 2Hz (12 Hz in this subject) and three stimulation intensities (1, 3, and 5). Top (intensity level 5): phase clustering was revealed by relatively high normalized Shannon entropy ($\tilde{\rho }$ = 0.0742). Bottom (intensity level 1): outside the Arnold tongue, Δ phase angles between EEG and stimulation signal are equally distributed in the phase plane. The normalized Shannon entropy $\tilde{\rho }$ as the quantitative measure is relatively low (0.0047).

To shed more light on this debate, here we used two physical concepts to systematically investigate the underlying mechanism of SSVEPs: the Arnold tongue and intermittency of phase locking (Pikovsky et al., 2003; Figure 1B shows a schematic Arnold tongue from synthetic data).

The Arnold tongue predicts the degree of synchronization (entrainment) of an oscillator coupled to a rhythmic driving force, depending on two parameters: the amplitude of the driving force (here: light intensity) and driving frequency. With a driving frequency that approaches the intrinsic frequency (here: IAF), entrainment is more likely to occur. With increasing intensity, the window of entrainment widens around the intrinsic frequency, allowing more distant stimulation frequencies to entrain the intrinsic oscillator compared to weaker stimulation intensities. This prediction would result in a triangular shaped area of entrainment when plotted as a function of driving intensity and frequency (green area in Figure 1B). At the border of this triangular shaped Arnold tongue (blue shaded area of Figure 1B) entrainment is intermitted by uncoupled time periods, so called phase slips. In other words, the intrinsic oscillator is coupled to the external stimulation phase for certain time periods, until, during constant prevailing stimulation, the internal oscillator slips back to the intrinsic frequency until it is again driven by the external stimulation. As opposed to the superposition of ERPs, the Arnold tongue predicts a non-linear response (Regan, 1982) that clearly identifies entrainment (Pikovsky et al., 2003).

Superposition of ERPs is described as processing of single events, independent of ongoing brain oscillations. Thus, we hypothesize that phase coupling should be independent of the regularity of the inter-stimulus interval at which the flicker is presented. Subjects were stimulated with 35 different conditions: five different light intensities and seven different frequencies, which were distributed around the IAF in steps of 1 Hz. Each of these rhythmic stimulation sequences was preceded by a jittered frequency with the same intensity and average number of flashes per second as the subsequent rhythmic condition. If superposition could indeed explain the SSVEP, the phase locking pattern of the 35 jittered sequences would not differ from that of rhythmic stimulation. Furthermore, a triangular entrainment region as predicted by the Arnold tongue would not be expected.

For the rhythmic stimulation conditions, phase locking was found to increase with increasing stimulation intensity as well as when the stimulation frequency approached the IAF, resulting in the triangular shaped pattern that is predicted by entrainment. Inside the triangular shape of the Arnold tongue, we found the rhythmic stimulation to show significantly stronger phase locking compared to the jittered conditions. Outside the Arnold tongue, the two hypotheses (superposition and entrainment) were indiscernible, as they both predict that no entrainment would occur, and here we found that phase locking showed no significant difference.

For the first time, we systematically compared the EEG of rhythmic and jittered stimulation with regard to the two hypotheses of the mechanism underlying SSVEPs: superposition of ERPs and entrainment of oscillations. We found that rhythmic—but not jittered—stimulation is capable of entraining ongoing brain oscillations.

## Materials and methods

### Participants

Thirty students from the University of Oldenburg were recruited (age 24.8 ± 3.4 years, 8 male subjects) for the study. All subjects gave written informed consent before their participation. Two subjects' data sets had to be discarded due to technical errors. Participants in the remaining sample reported no vision deficits, psychiatric disorders, epilepsy in family history or febrile convolutions during childhood. According to the Edinburgh handedness inventory (Oldfield, 1971), five of the 28 subjects were left-handed. The experimental protocol was approved by the ethics committee of the University of Oldenburg and was conducted in accordance with the Declaration of Helsinki.

### Design and procedure

Subjects were seated in a dark sound-attenuated EEG chamber 60 cm away from an LED light, which was embedded into a black background, adjusted at individual eye-level. The experiment consisted of two parts. In the first part, EEG was recorded during resting state for 2 min in the dark. From this data, the IAF was determined (see below).

**Figure 2:**
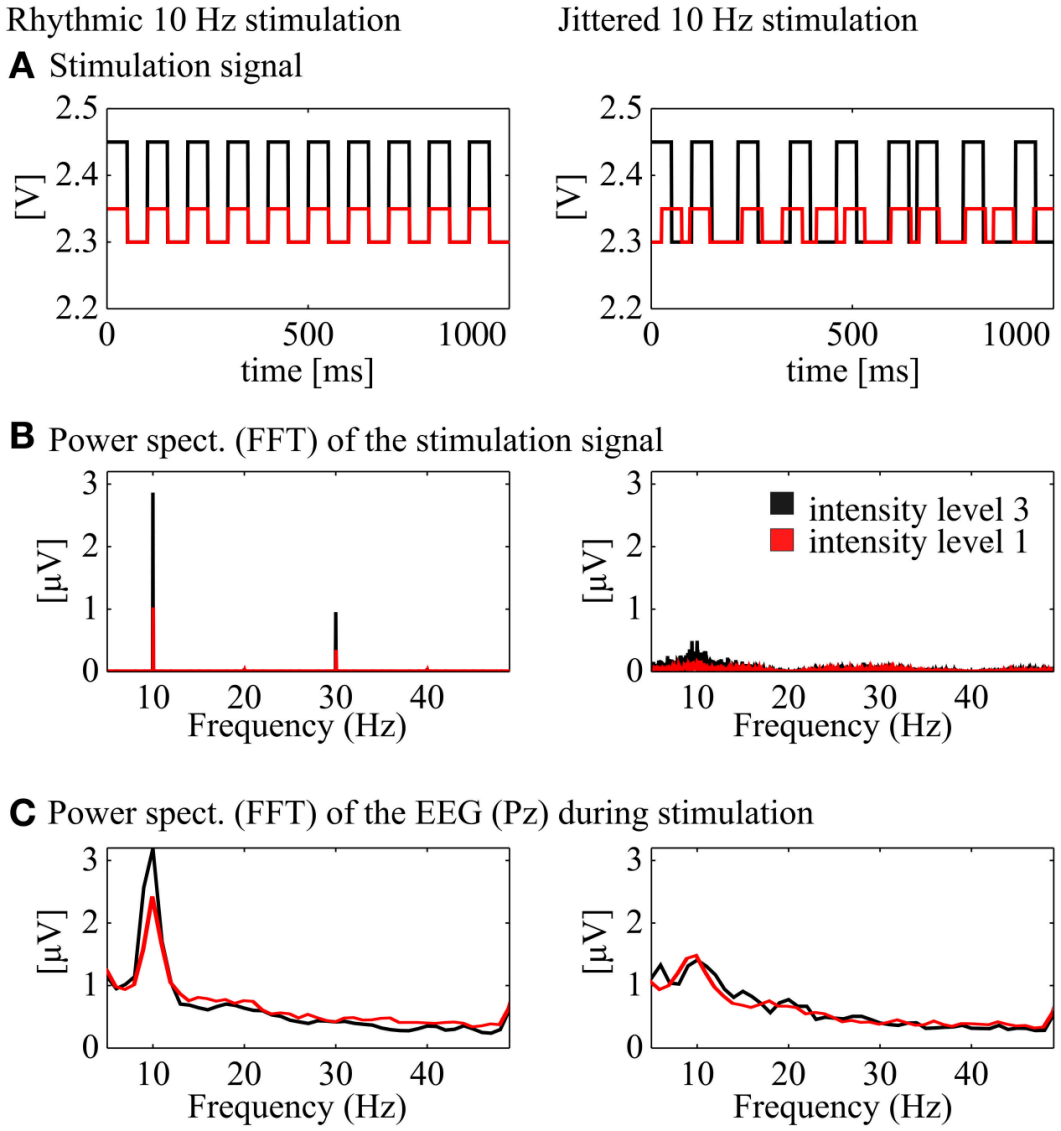
**Demonstration of the stimulation signal and the corresponding power spectra of the stimulation and the EEG signal. Left:** rhythmic stimulation at 10 Hz, **right:** jittered stimulation at 10 Hz (20, 35, 50, 65, or 80 ms ISI). **(A)** Stimulation signal in Volt [V] for one exemplary second from the 30 s stimulation interval at medium intensity (black) and low intensity (red; rhythmic and jittered, respectively). **(B)** Power spectra of the two signals above (FFT, Hanning window, 1 s pieces, 26 s, center of the stimulation interval). **(C)** Power spectra (FFT, Hanning window, 1 s pieces, 26 s) of the corresponding EEG signal at Pz.

In the main experiment (after resting state measurement), participants visually fixated onto an LED which flickered at five different light intensities and seven different frequencies. The flicker was elicited in the LED by driving it with a square wave voltage (cf. Figure 2, Box 1A–C). We choose a square wave instead of a sine, as the linear luminance increase of the LED was located in a low voltage range, insufficient for covering the potential area of the Arnold tongue. In other words, it was not possible to drive the LED in a perfect sine using the intensities necessary to entrain the internal oscillations at multiple light intensities. This is, however, not a restriction, as Dreyer and Herrmann (2015) showed that also sine wave stimulation produces harmonics in the EEG spectrum. The five stimulation intensity levels altered in luminance between 1.42 cd/m^2^ (minimum voltage, close to visual threshold) and either 8.34, 52.8, 326, 1756, or 7158 cd/m^2^ (maximum voltage for the five different intensities), which corresponds to a linear increase in voltage. These intensities will henceforth be referred to as intensities 1–5. The stimulation frequency was varied from −3 to +3 Hz around the IAF, determined in the preceding resting state measurement. The LED was operated by a NIDAQ device (National Instruments Data Acquisition, National Instruments Germany GmbH, Munich, Germany), which converted the digital output generated by a script written in MATLAB R2012b (The MathWorks Inc., Natick, MA, USA) into an analog voltage.

Conditions were created with the 5 × 7 intensity–frequency combinations and each condition was presented to the participants for 30 s, followed by a 2 s pause. Preceding each single condition, a 30 s stimulation of jittered flickering light was presented, where instead of a rhythmic flicker inter stimulus intervals (ISIs) of the square wave were jittered with a maximum of ±60% (ISI from the subsequent rhythmic condition, e.g., 50 ms in the 10 Hz condition x1.6, x1.3, x1, x0.7, x0.4, resulting in 20, 35, 50, 65, or 80 ms). The ISIs were randomized with a uniform distribution over the whole stimulation period and the same ISIs never appeared twice in a row. Figure 2A shows an exemplary 1 s sequence of a rhythmic and a jittered stimulation interval. Below (Figure 2B), the averaged power spectrum (FFT, Hanning window, 1 s pieces) of the stimulation signal shows a clear peak at 10 Hz for the rhythmic stimulation (plus power at the harmonic frequencies). The arrhythmic character of the jittered stimulation is revealed by a flat noisy spectrum with less power at the stimulation frequency than the rhythmic stimulation at around 10 Hz. These characteristics are found likewise for the EEG spectrum at Pz (FFT, Hanning window, 1 s data pieces). The jittered stimulation was used as a control for the rhythmic condition. The order of the stimulation-jitter pairs was randomized between subjects. The main experiment was subdivided into three blocks of 12.4 min with pauses of individual length between the blocks. The duration of the whole procedure including EEG cap preparation was ~90 min.

### EEG measurement

**Figure 3:**
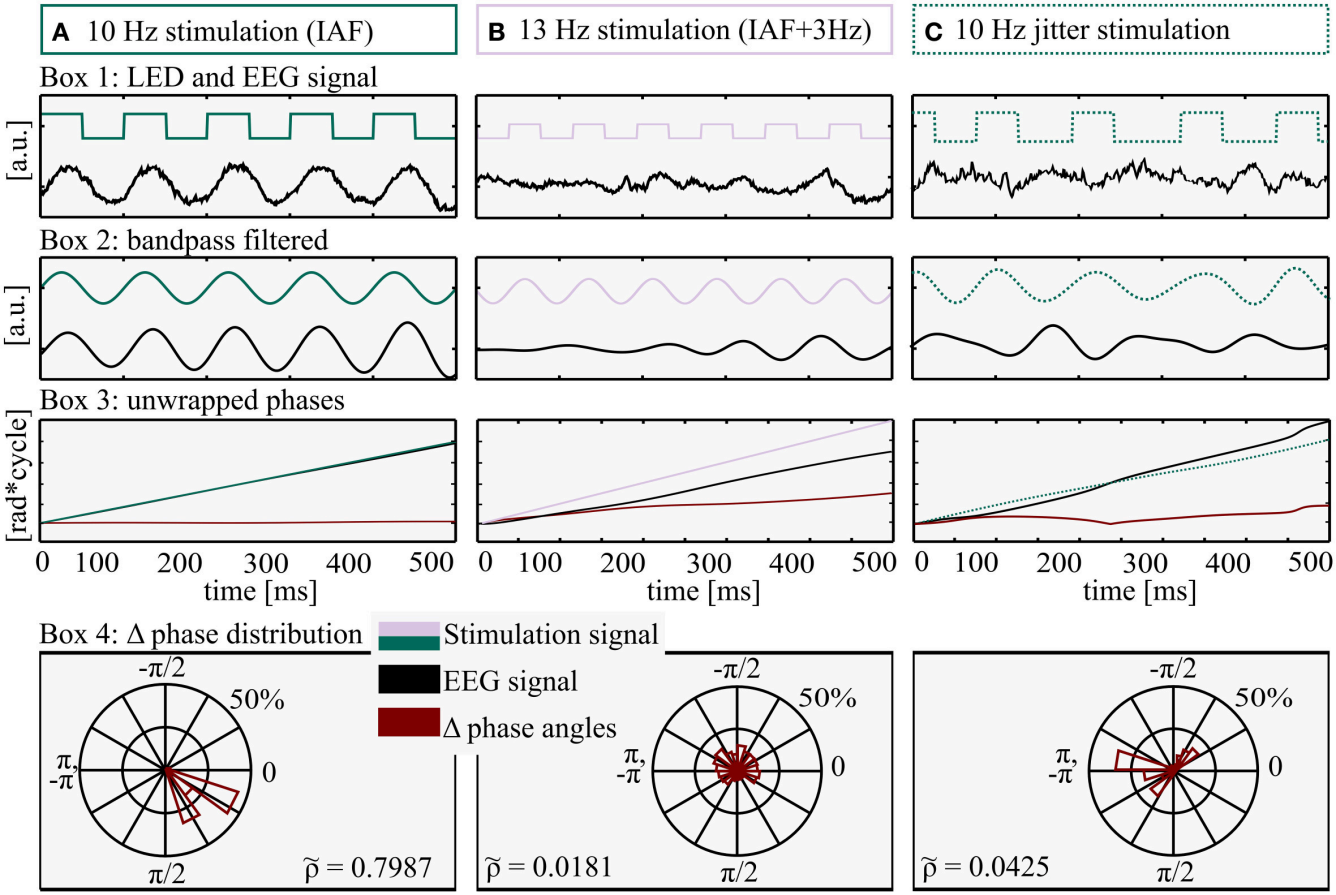
**Method for determination of Shannon entropy**. All processing steps were performed on three exemplary data pieces (500 ms) from three different conditions: **(A)** Stimulation intensity 5 (max), frequency: IAF (individual alpha frequency, 10 Hz here), **(B)** Stimulation intensity 1 (min), frequency: IAF+ 3 Hz, 13 Hz here and **(C)** Jittered stimulation at 10 flashes per second. Box 1: LED stimulation signal (square wave, green/lilac) and EEG raw data (black). Box 2: filtered LED stimulation signal (green/lilac) and EEG (black). Box 3: unwrapped phase angles of LED stimulation signal (green/lilac) and EEG data (black). The red line depicts the time course of Δ phase (EEG–LED stimulation). Box 4: distribution of wrapped Δ phase angles. The almost horizontal course of Δ phase angles in Box 3A (red graph) is approaching a Dirac-like distribution, quantified by a relatively high Shannon entropy ($\tilde{\rho }\;=$ 0.7987), whereas the rather uniform distributions and a lower Shannon entropy ($\tilde{\rho }\;=$ 0.0181 and 0.04325) in Box 4B and 4C reflect a drift of the two phase angles (LED stimulation and EEG signal) and as a consequence an ascending Δ phase angle over time (Box 3B, red line).

The EEG was measured from four sintered Ag–AgCl electrodes at O1, Oz, O2, and Pz, mounted in an elastic cap (Easycap, Falk Minow, Munich, Germany) with an extended 10–20 system layout, referenced to the nose. For further analysis steps, only Pz was chosen as the alpha oscillator has been described to show its maximum peak in a power spectrum in that region (Ergenoglu et al., 2004). Furthermore, averaging of multiple electrodes might reduce the overall effect due to phase shift between the channels. The ground electrode was placed at FPz, impedances were kept below 10 kΩ. EEG was recorded using a 16-bit Brain Amp DC amplifier and Brain Vision Recorder software (Brain Products GmbH, Gilching, Germany). An online band pass filter of 0.016–250 Hz and a sampling rate of 5000 Hz were used. EEG was amplified in the range of ±16.384 μV at a resolution of 0.5 μV/bit. Stimulus markers and EEG data were stored digitally for further offline analysis. Raw exemplary EEG data is displayed in Figure 3 (Box 1).

### Data analysis

Data were down-sampled to 1000 Hz and filtered using a windowed since type I linear phase FIR filter (band pass: IAF ± 3.5 Hz, filter order: 6002) and did not induce a phase shift in the alpha range (6–14 Hz). The identical filter was used for the stimulation signal and the EEG data (Widmann and Schröger, 2012). Filtered EEG data are shown in Figure 3 (Box 2). For each condition, the first and last 2 s were discarded, since the steady-state condition has to build up and to avoid edge effects (Halbleib et al., 2012). All analysis steps were performed in MATLAB R2012b (The MathWorks Inc., Natick, MA, USA) and EEGLAB 11.0.4.3 (Delorme and Makeig, 2004).

#### Determination of individual alpha frequency

Prior to the experiment, 2 min of resting state EEG at the Pz electrode were recorded in a dark and sound-attenuated EEG chamber with eyes closed. A power spectrum was calculated using Fast Fourier transformation on 1 s data pieces, windowed with a rectangular taper. The induced spectra were averaged. The IAF was then defined as the maximum peak within the alpha range (9–11 Hz) in the eyes closed condition to remain within a comparable stimulation range. Ten of the 28 subjects showed no clear peak within this range. These subjects were assigned a standard IAF of 10 Hz, resulting in a mean center stimulation frequency of 9.96 ± 0.42 Hz. Furthermore, the 9 Hz subjects had to be excluded from the analysis (three subjects) as the harmonics of the IAF—3 Hz stimulation (= 6 Hz) lay within the band pass window, leaving 25 subjects for the analysis.

#### Phase detection

To classify the degree of entrainment, we determined the phase-locking of the stimulation and the EEG signal. Each step of the analysis is depicted in Figure 3 for two rhythmic conditions as well as one jittered condition. We used the Hilbert transform function (Matlab) on electrode Pz, because it shows the maximum alpha amplitude (Ergenoglu et al., 2004). To calculate the difference of the phase angles of the EEG data and the stimulation signal (Δ phase angles), the unwrapped phase angles of the Hilbert transform (Figure 3, Box 3A,B) were subtracted (EEG phase minus stimulation phase) and wrapped to the range of −π to π (Figure 3, Box 4). To quantify entrainment for each subject and condition, the distribution of Δ phase angles was quantified in a range between uniform distribution (complete independence of the two signals) and a Dirac-like distribution (perfect phase synchronization). Therefore, the normalized Shannon entropy (Tass et al., 1998) was determined for the Δ phase angles at each time point, condition and subject, in the range of −π to π to quantify the phase locking over time.

The normalized Shannon entropy $\tilde{\rho }\;$is defined as:

$$\begin{array}{l} \tilde{\rho }=\;\frac{S_{max}-S}{S_{max}}\text{with S}=\;-\sum_{k=1}^{N}\;p_{\text{k}}\;\ln\;p_{k}\text{ and }S_{max}=\ln\;N, \end{array}$$

where *N* is the number of bins (*N* = 80), *p*_*k*_ is the probability of the *kth* bin and S is the classic Shannon entropy of a fixed window between −π to π. The normalized Shannon entropy can take values between $0\leq \tilde{\rho }\leq 1$, where $\tilde{\rho }=0$ corresponds to a uniform distribution and $\tilde{\rho }=1$ to a Dirac-like distribution (Tass et al., 1998). At a glance, high entropy S (low normalized Shannon entropy $\tilde{\rho }$) reflects a rather random distribution of Δ phase angles over the bins of a polar histogram, indicating no entrainment (Figure 3, Box 4B), while low classic Shannon entropy (high normalized Shannon entropy $\tilde{\rho }$) results from a rather constant phase coupling (i.e., entrainment, Figure 3, Box 4A). Perfect coupling gathers all values within a single bin (Dirac-like distribution).

The normalized Shannon entropies were calculated for all subjects and conditions. We predicted that in case of entrainment $\tilde{\rho }$ should increase with increasing stimulation intensity and with decreasing distance from IAF. Thus, conditions were arranged in a two-dimensional plane as a function of intensity (five levels) and frequency (seven levels).

#### Intermittency

When the intrinsic brain oscillations are entrained by the driving force, the Δ phase angles between EEG and driving force are close to zero and, as a consequence, are considered a phase plateau. This is the case when frequency and intensity are chosen such that they are located inside the triangular shape of the Arnold tongue. Outside the Arnold tongue, the EEG is not synchronized to the driving force and Δ phase angle constantly increases over time. Intermittency is defined as an alternation of phase plateaus and phase slips (see below) and is observed at the border zone of entrainment regions. Phase plateaus are horizontal periods of the unwrapped Δ phase angles when plotted over time (see exemplary data of Figure 1A, marked by a ^*^). Plateaus show increasing length with increasing driving intensity and decreasing frequency distance from IAF. To test for the effects of intensity and frequency, plateaus were identified for each sampling point by determining the slope (±100 samples). A gradient of 0 ± 0.005 was defined as plateau and the duration was then counted. This gradient was implemented to account for noise in the data. Ten plateaus were therefore classified as such by visual inspection. The mean standard deviation of these 10 random plateaus was taken as deviation for the plateau classifier algorithm. Stronger gradients of Δ phase angles were considered phase slips. Intermittency was quantified as the maximum plateau duration per condition and was averaged across subjects. When intermittency occurs, the characteristics of the driving oscillator (intensity and frequency) neither describe conditions outside the Arnold tongue, where the two phases linearly drift apart (constant phase slip), nor inside the triangular shape of the Arnold tongue, where the two oscillators are coupled (constant plateau).

### Statistics

#### Normalized shannon entropy

In the first step, the normalized Shannon entropies $\tilde{\rho }$ of the Δ phase angles for the rhythmically stimulated conditions were tested against $\tilde{\rho }$ of the Δ phase angles for the corresponding jittered conditions. Because data failed to be normally distributed, non-parametric Mann-Whitney *U*-tests (Fay and Proschan, 2010) were applied. From the resulting *z*-values the effect sizes (*r*) were calculated:

$$\begin{array}{l} r=abs\left(\frac{z}{\sqrt{N}}\right),\text{ with}\;\text{N}=\text{50} \end{array}$$

(Fritz et al., 2012).

For the superposition hypothesis, we would expect brain responses to be independent of the driving rhythm. The resulting *p*-values were corrected for false discovery rate (FDR) at 0.001, 0.01, and 0.05. This procedure was applied in order to control for Type I errors by sorting the *p*-values, then correcting for multiple comparisons starting with the smallest *p*-value (Benjamini and Hochberg, 1995).

**Figure 4:**
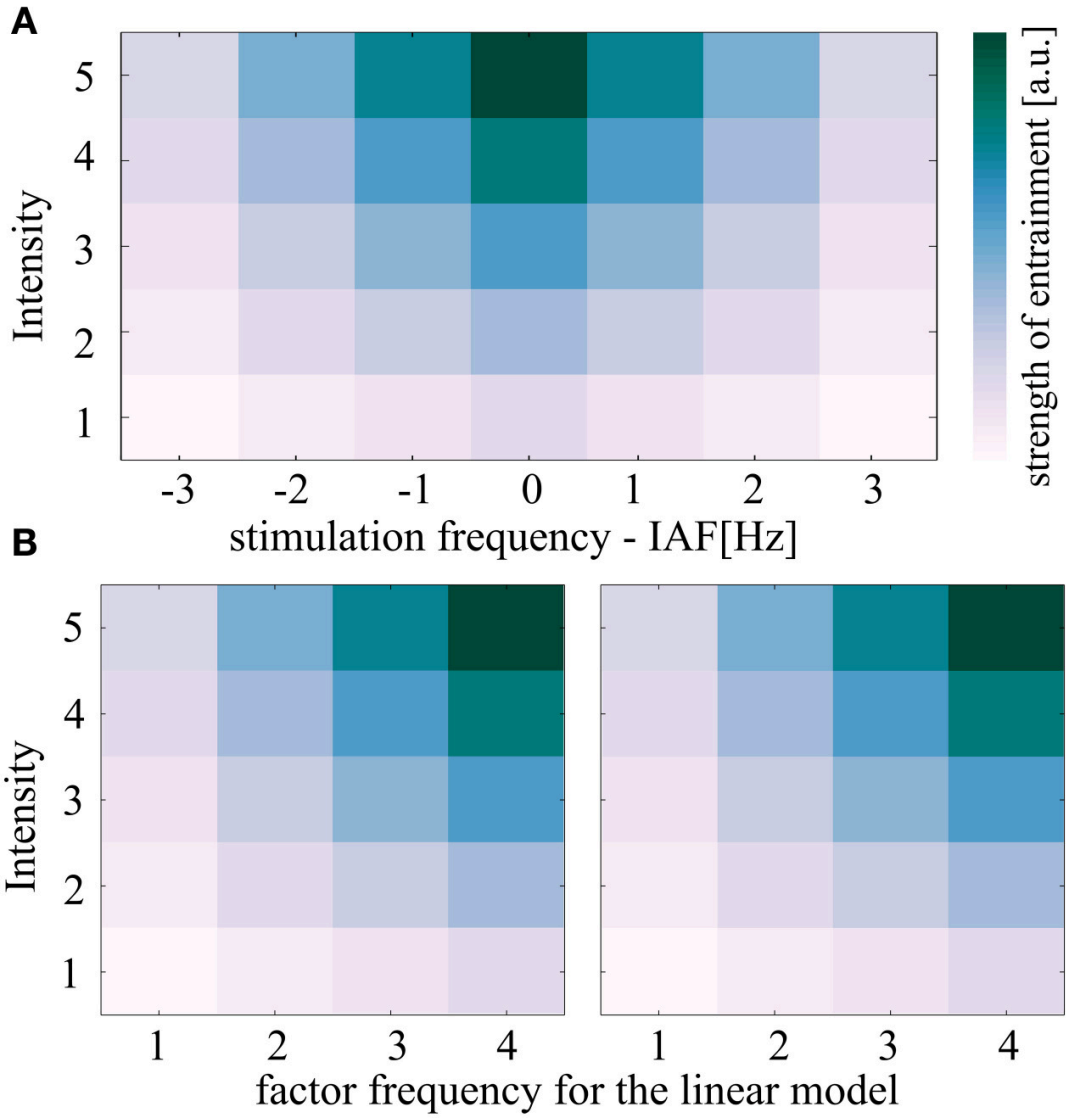
**Description of the linear model used to test for the predicted shape of the Arnold tongue**. **(A)** The theoretical concept of entrainment predicts the depticted triangular region of synchronization referred to as Arnold tongue. Both independent variables intensity and frequency are expected to correlate with the entrainment measure (here: either normalized Shannon entropies or 90% of plateau durations). **(B)** If the Arnold tongue is split in two halves and the right half is flipped in frequency, a linear model can test for the triangular shape. Whenever the two coefficients for intensity **(A)** and frequency **(B)** in the linear model are both positive such a triangular pattern results (unless the coefficient c which represents the interaction is negative). The shown plots were achieved by setting the parameters a and b of the linear model to 1. Parameter c was set to zero.

**Figure 5:**
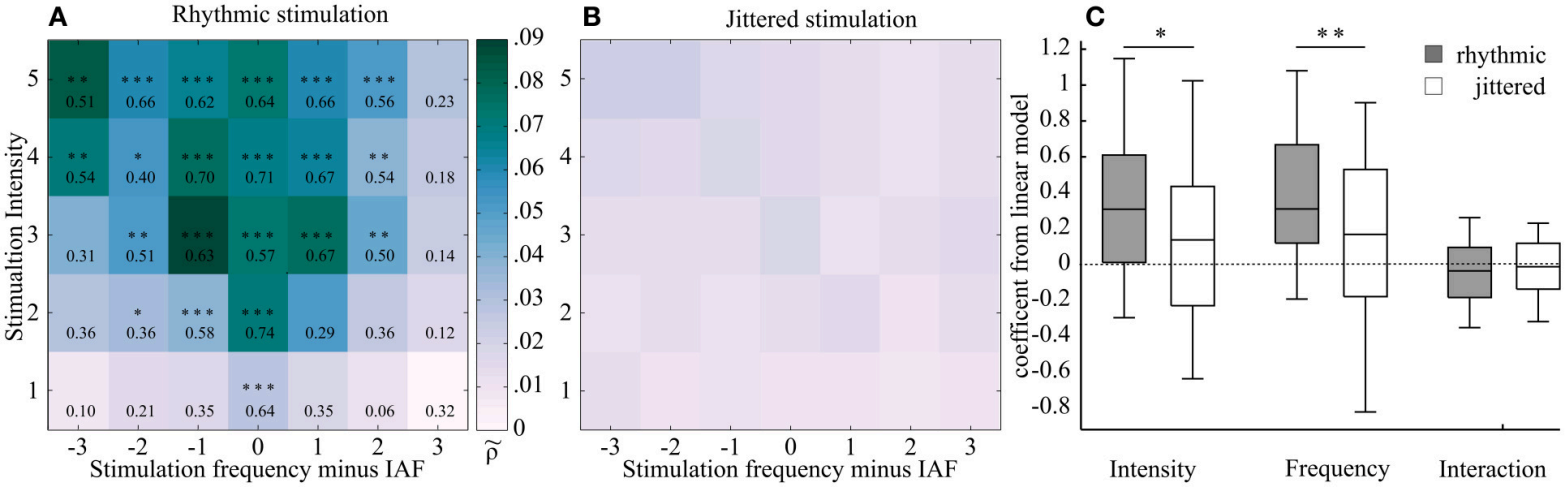
**Two two-dimensional planes showing averaged Shannon entropies (25 subjects) as a function of frequency and intensity of the external stimulation, centered at individual alpha frequency (IAF). (A)** Rhythmic stimulation. Stronger phase locking is shown in darker shading (higher $\tilde{\rho }$-value). This region was found around IAF and widens with increasing intensity, resulting in a triangular shape. This shape corresponds to the hypothesized Arnold tongue. Effect sizes (r) from a Mann-Whitney *U*-test show the comparison between the rhythmic condition and the respective jittered condition. Asterisks mark level of significance after FDR correction (^***^p < 0.00008, ^**^p < 0.0004, ^*^p < 0.005). **(B)** Jittered stimulation. No such triangular shape was observed in the jittered condition. **(C)** Box plots of the coefficients of the linear model, fitted to rhythmic (gray) and jittered (white) stimulation of the normalized Shannon entropies subjects-wise for the two sides of the triangle (see Figure 4 for model description). A Mann-Whitney-*U***-**test was performed to test for differences between jittered and rhythmic stimulation for the dependent variables frequency and intensity. Both factors but not the interaction term were found to differ significantly (Intensity: *r* = 0.19, *z* = 1.92, *p* < 0.05; Frequency: *r* = 0.27, *z* = 2.66, *p* < 0.01; Interaction: *r* = 0.08, *z* = −0.78, *p* = 0.78). In the plot, the middle of the box represents the median. The box represents the 95% confident interval and the whiskers the 25th and 75th percentiles.

In the second step, the resulting shape of the pattern in the two-dimensional plane was tested. Therefore, $\tilde{\rho }$-values were z-standardized subject-wise to guarantee normal distribution and reduce inter-subject variance. Then, a linear model (see below) was fitted to the normalized Shannon entropies. Based on the hypothesized pattern of the Arnold tongue, we expect the normalized Shannon entropy values to increase with increasing stimulation intensity. For the factor frequency, we expect a positive impact on the normalized Shannon entropies for the left half of the triangular shape, the closer the stimulation frequency was to the IAF (scaled from Δ3 Hz = 1 to Δ0 Hz = 4), the higher the $\tilde{\rho }$ (= stronger entrainment). As the factor frequency shows non-linear characteristics in the Arnold tongue (the strongest entrainment is predicted for the center of the Arnold tongue), the 5 × 7 (intensity × Frequency) data structure as depicted in the two-dimensional plane of the Arnold tongue in **Figures 5A,B** was mirrored to the left side to gain linear characteristics. The linear model is depicted in Figure 4.

The model was then fitted to each of the 25 subjects and for both sides of the Arnold tongue (Figure 4B) separately, resulting in five stimulation intensities and four frequencies per linear model. The 2 × 25 coefficients (sides of the predicted Arnold tongue x subjects) were concatenated into one data matrix for the independent variables intensity and frequency as well as the interaction term, respectively, resulting in 3 × 50 coefficients (Intensity, Frequency and Interaction term x coefficients of two sides for 25 subjects).

$$\begin{array}{l} y=a*int+b*freq+\;c*int*freq \end{array}$$

This procedure was repeated for both the rhythmic and the jittered stimulation. The 3 × 50 coefficients of the rhythmic stimulation condition were then compared to the respective jittered coefficient, using a one-sided Mann–Whitney-U-test (Fay and Proschan, 2010) as data failed to show normal distribution using a Shapiro–Wilk-test (Royston, 1993). Effect sizes *r* were calculated with *N* = 100 following the above described formula (Fritz et al., 2012).

#### Plateaus

Again the above described linear model was fitted to the 90 percentile of the sorted plateau durations for the rhythmic condition. The two-dimensional data matrix (intensity × frequency) was therefore reshaped as described above to include the factor frequency as a linear independent variable (see Figure 4) and plateau durations were z-normalized before fitting the linear model. Then, *t*-tests were performed for the three coefficients (intensity, frequency, and the interaction term) to test against zero. If it is true that plateau duration (as a measure of phase coupling over time) increases with increasing stimulation intensity, we expect the coefficients to be significantly greater than zero. Furthermore, if it was true that frequency increase (for the left half of the triangle, mirrored for the right half) has a positive impact on plateau duration, then we expect the coefficient to be significantly greater than zero. The effect sizes *r* were calculated by the following formula:

$$\begin{array}{l} r=\;\sqrt{\left(\frac{t^{2}}{t^{2}+df}\right)} \end{array}$$

(Cohen, 1988).

## Results

The paradigm was designed in a way that allowed the comparison of different fields in the hypothesized plane of the Arnold tongue. Subjects were stimulated with a rhythmically flickering light at five intensity levels and at seven frequencies, centered at the IAF in steps of 1Hz, resulting in 35 conditions. Each condition was presented for 30 s, with a preceding jittered sequence (also for 30 s).

The intrinsic frequency of the parietal brain oscillations is defined as the IAF, whose most prominent peak in the individual power spectrum is located in the parieto-occipital cortex during resting state (Berger, 1929). Following the concept of the Arnold tongue (Pikovsky et al., 2003), we expect an increased likelihood for a driving frequency close to the IAF to entrain the internal oscillator as compared to frequencies more distant to IAF. As a second factor, brighter light flicker should result in stronger phase coupling compared to weaker stimulation intensities. If these dependencies of stimulation frequency and light intensity can be shown, entrainment but not superposition of ERPs is the most likely explanation for SSVEPs.

Two measures were applied to quantify the level of entrainment, plateau duration of phase locking over time (the longer the plateau, the stronger the entrainment) and the overall phase locking, defined as the normalized Shannon entropy (the less entropy (larger value) the stronger the entrainment, because strong phase locking = entrainment). Note, that a larger value of normalized Shannon entropy indicates less entropy and stronger phase coupling, which is interpreted as stronger entrainment.

When depicting the quantified Δ phase angle distributions (normalized Shannon entropy $\tilde{\rho }$ of the phase of the EEG signal minus the visual flicker phase) as a function of frequency and intensity, a resulting triangular shape would reveal entrainment of an oscillator, as defined by the physics principle of the Arnold tongue. Additionally, the entrained areas of the Arnold tongue (inside the triangle) are expected to show a low phase difference (strong directionality of Δ phase angle distributions). The effects of stimulation intensity and frequency were tested and the shape of the resulting plot was compared to the triangular shape predicted by the Arnold tongue. Next, the plateau durations and the normalized Shannon entropies were compared to the jittered (arrhythmic) conditions.

### The normalized shannon entropy as a measure of entrainment

At the IAF, all five stimulation intensities showed constant phase locking. This phenomenon is depicted by a slope close to zero (Figure 1A, first plot), reflecting strong phase locking, i.e., high normalized Shannon entropy (= strong entrainment). In the schematic Arnold tongue from synthetic data in Figure 1B the expected position in the two-dimensional plane are indicated. On the contrary, at greater distance from IAF outside the Arnold tongue, no entrainment was expected. This was reflected in the phase of the EEG signal by (almost) unlocked phases, independently of the stimulation intensity, as shown for the exemplary dataset in Figure 1A, fourth plot. Thus, for these two frequency conditions (at IAF and far away from IAF) phase locking was found to be robust over time, representing entrainment by a slope close to zero in the first case and a linearly ascending slope, reflecting no entrainment in the second case.

At intermediate distance from the IAF (Δ 2Hz), the effect of intensity becomes particularly clear: at lower intensities, the phase angles of the EEG signal and the stimulation signal appeared to be similarly unlocked as in the Δ 3Hz condition, as reflected in the constant increase of Δ phase angles (cf. lilac graph in Figure 1A, third plot). With increasing stimulation intensity, the slopes of Δ phase angles show a weaker linear increase, indicating stronger phase locking (entrainment; cf. blue and green graph in Figure 1A, third plot).

Figure 1C depicts how the slopes of Δ phase angles translate to the distribution over the unit cycle, which was then expressed as normalized Shannon entropy $\tilde{\rho }\;$for three intensities [minimum (1), mediate (3), and maximum (5)] of the exemplary data at Δ2 Hz. The distribution of Δ phase angles was determined to quantify the phase locking over time. While the low intensity levels are reflected by a uniform distribution of Δ phase angles (outside the Arnold tongue), the distribution becomes more directional with increasing stimulation intensity (toward areas inside the Arnold tongue). This directionality is expressed by $\tilde{\rho }$, which increases in the same (${\tilde{\rho }}_{1}=0.0047,\;{\tilde{\rho }}_{3}=0.0085,\;{\tilde{\rho }}_{5}=0.0742$).

The $\tilde{\rho }$-values, arranged in the two-dimensional plane as a function of intensity and frequency, resulted in a triangular shape, showing increasing normalized Shannon entropy (increased synchronization) at and around the intrinsic frequency (IAF, Figure 5). The triangular shape was reflected by the following statistical tests:

First, the distributions of Δ phase angles from rhythmic stimulation were compared to the jittered conditions. Each of the 35 rhythmic stimulation conditions was preceded by its corresponding jittered condition with a mean frequency of the subsequent rhythmic stimulation frequency during the experiment. The intensity was also matched.

The effect sizes were found to increase gradually toward the IAF and with increasing stimulation intensity. Moreover, the normalized Shannon entropy $\tilde{\rho }$ was found to significantly differ from the jittered condition only within a triangularly shaped pattern around the IAF, as revealed by Mann–Whitney *U*-test statistics, which were performed on each rhythmic–jitter pair. The significance level was false discovery rate (FDR)-corrected (*p* < 0.00008, *z* ≥ 3.96, *r* ≥ 0.56; *p* < 0.00044, *z* ≥ 3.54, *r* ≥ 0.50; *p* < 0.00490, *z* ≥ 2.55, *r* ≥ 0.36). *z-*values were transformed to effect sizes (*r*), which are shown together with the *p*-values for each condition in the respective field of Figure 5A. Figure 5B shows the two dimensional plane of normalized Shannon entropies for the jittered condition.

In order to test for the triangular shape in the two-dimensional plane, a linear model with the independent variables intensity and frequency was fitted to the data (see Materials and Methods Section and Figure 4 for details) of each individual subject. The linear model was fitted to the normalized Shannon entropies from both, rhythmic and jittered stimulation conditions.

The coefficients for the rhythmic stimulation of both variables—intensity and frequency—were found to significantly differ from the jittered stimulation in a one-sided Mann-Whitney *U*-test (Intensity: *r* = 0.19, *z* = 1.92, *p* < 0.05; Frequency: *r* = 0.27, *z* = 2.66, *p* < 0.01; Interaction: *r* = 0.11, *z* = −0.78, *p* = 0.78). The box plot in Figure 5C shows the distribution of the coefficients. As explained in the Materials and Methods Section and shown in Figure 4, the positive coefficients for intensity and frequency imply a triangular shape of the region of entrainment and are thus in line with an Arnold tongue.

### The plateau duration as a measure of entrainment

Besides the slope of Δ phase angles, the intermittency of phase locking is an important criterion to identify synchronization (Pikovsky et al., 2003). The phenomenon, known from entrainment of isolated physical oscillators, describes disturbed phase locking (phase slips) during constant prevailing stimulation. While a linear addition of single responses (superposition hypothesis) is expected to result in a constant EEG response, synchronization caused by entrainment can take different states of phase locking over time. The duration of the respective states depends on the frequency and intensity of the driving oscillator, as described by the Arnold tongue. Phase slips, which result from drifting apart of the two phases, are expected to be extended in time and to appear more frequent with decreasing coupling of the two oscillators (Tass et al., 1998; Pikovsky et al., 2003).

Intermittency becomes particularly obvious at the border of the Arnold tongue (light green to blue shaded area in Figure 1B). The data of the exemplary subject shows phase locking plateaus at higher stimulation intensities (Figure 1A, third plot, green curves; exemplary period marked by the asterisk) with intermittent drifting apart of the two oscillators (phase slips, one exemplary period is marked by the arrow).

Phase locking plateaus were quantified in order to test for the predicted effects of intensity and frequency. Therefore, the linear model was fitted to the z-normalized 90 percentiles of plateau durations subject wise. We found the independent variable intensity to significantly influence the plateau duration by testing the referring coefficients of the linear model using *t*-tests [95% CI [0.46, 0.21], 0.17 ± 0.07, *t*_(49)_ = 3.18, *p* < 0.01, *r* = 0.41]. The coefficients for the factor frequency were also found to significantly differ from zero [95% CI [0.30, 0.52], *t*_(49)_ = 7.68, *p* < 0.001, *r* = 0.74]. No significant effects were found for the interaction term: 95% CI [−0.02, 0.05], *t*_(49)_ = 0.67, *p* = 0.50, *r* = 0.10.

## Discussion

The present study reveals evidence that the SSVEP reflects entrainment. Our findings support the assumption that a visual flickering light stimulation can be applied to study the functional relevance of brain oscillations.

Sensory rhythmic stimulation is broadly accepted as an eligible tool to produce entrainment. Mathewson et al. (2010) stimulated subjects with a 12.1 Hz rhythmic flickering light and presented a masked target in phase after stimulation offset. The number of rhythmic flicker stimuli varied between 1 and 8 and was shown to positively correlate with in-phase target detection. Likewise, as a consequence of 10 Hz rhythmic stimulation Spaak et al. (2014) found the detection rate to significantly depend on stimulation phase. In their study, a 10 Hz flicker was presented to one hemifield; the other hemifield was stimulated with a flicker stream with jittered inter-flash intervals. Phase effects were found solely for the rhythmic condition. In both studies, entrainment is inferred as the fundamental mechanism of the produced SSVEP. To clearly trace back the described behavioral changes after visual rhythmic stimulation, it is however necessary to provide evidence that SSVEPs indeed reflect entrained oscillations.

The interaction of the rhythmic visual stimulation and the targeted oscillation has recently been questioned (Capilla et al., 2011; Keitel et al., 2014), which highlights the particular relevance and the timeliness of this investigation. By probing two physics concepts that unambiguously define entrainment: intermittency of phase locking and the Arnold tongue, we systematically tested the two hypotheses suggested to reflect the fundamental mechanism of SSVEPs, superposition and entrainment.

A non-rhythmic (jittered) stimulation is unable to entrain the intrinsic frequency (Parkes et al., 2004; Capilla et al., 2011). Thus, we predicted a similar pattern for the phase locking of the jittered condition and the rhythmic stimulation, assuming the superposition hypothesis was true. This was not the case. Our data were in line with the entrainment hypothesis: the normalized Shannon entropies of the rhythmic condition tested against those of the jittered condition showed an increasing effect size with increasing intensity and decreasing frequency distance to IAF in the condition wise comparison (Mann-Whitney-*U*-tests). At the maximum distance from IAF (Δ3 Hz here) and at low stimulation intensity, the two signals revealed non-distinctive phase coupling with lower effect sizes and non-significant differences between jittered and rhythmic stimulation. The fitted linear model revealed significance for the observed triangular shape of the normalized Shannon entropies, as both factors-frequency and intensity-showed a positive value (Figure 5C). This can only result in the predicted triangular pattern and was significantly different from the jittered stimulation condition.

The areas outside the Arnold tongue are characterized by relatively lower stimulation intensity and/or a driving frequency that is more distant to the intrinsic frequency (IAF). These characteristics are ineligible to entrain the internal oscillator. Hence, outside the Arnold tongue, stimulation responses are independent from the intrinsic alpha oscillation and thus rather resemble sequences of ERPs, as also predicted by the superposition hypothesis. Inside the Arnold tongue, however, the normalized Shannon entropy showed significantly stronger phase coupling (increased effect size r in a Mann-Whitney-*U*-test between jittered and rhythmic stimulation), which is interpreted as entrainment. Oscillations are entrained, such that the intrinsic frequency is shifted toward the driving frequency. As a consequence, only under these circumstances one can investigate the function of the intrinsic oscillations. de Graaf et al. (2013) found behavioral entrainment effects at 10.6 Hz, whereas effects disappear at 7.1 Hz and below as well as at 14.2 Hz, which is in accordance with the presently identified borders of the Arnold tongue at intermediate intensity.

Intermittency of phase locking reflects a non-linear process, where the oscillator is phase locked over certain periods, but then changes its phase during constant prevailing circumstances (Pikovsky et al., 2003). In the center of the Arnold tongue, in line with the prediction of this concept, these intermittent phase slips are rather unlikely to appear, as phase locking remains robust over the stimulation period (Figure 1A, plot 1). The method for plateau detection was designed in a rather conservative way, which explains why the maximum plateau duration for IAF entrainment did not span over the entire stimulation period, as inferred from Figure 1A, plot 1. Noise eventually interrupted the plateaus, but as the procedure was identical in all conditions, the relative comparison is unaffected by noise. While the normalized Shannon entropies reflect a relative measure that identifies the shape of the Arnold tongue when comparing different intensity-frequency stimulations, intermittency is a measure that provides evidence for entrainment on a single subject level. Therefore, in order to show entrainment, it is sufficient to examine the intermittency at a stimulation condition at the border of the Arnold tongue, between the non-significant normalized Shannon entropies (suggested area outside the Arnold tongue) and the conditions expected to lie inside the Arnold tongue (Figure 5A). The phase slips are expected to result from a transient uncoupling of the driving oscillator (light flicker), where the intrinsic oscillator temporarily rotates at IAF. Although alpha amplitudes fluctuate over time as they correlate with cognitive functions (Worden et al., 2000; Foxe and Snyder, 2011; Klimesch, 2012) it is rather unlikely that this would cause phase slips. The effect of attention should be distributed equally over conditions. Quite on the contrary, we found the plateau duration to reveal a shape that resembles an Arnold tongue. Both factors frequency and intensity were significantly greater than zero when fitting a linear model. As depicted in Figure 4, this can only result in a triangular shape on the 5 × 4 half of the Arnold tongue.

Despite the clear triangular shape as predicted by the concept of the Arnold tongue, the maximum values were not located at the highest stimulation intensity, but rather at intermediate intensity, which might be due to intensity saturation. Buchsbaum and Pfefferbaum (1971) showed a decreased ERP amplitude when an individual threshold intensity level was exceeded. In their study, the level was highly variable between subjects. This might also explain why we did not find significant effects for the interaction term in the linear models for normalized Shannon entropies and plateau durations.

The triangular shape of normalized Shannon entropies is slightly skewed to the left. This could be due to the fact, that participants were passively stimulated without performing a demanding task, as the alpha frequency has been shown to positively correlate with cognitive demands (Haegens et al., 2014).

The notion that SSVEPs are generated via the entrainment of an intrinsic brain oscillation also explains non-linear phenomena of SSVEPs that could not be explained by linear superposition of ERPs. For example, Herrmann (2001) demonstrated that the SSVEP in response to 80 Hz flickering light showed a clear 10 Hz oscillation. Superposition of ERPs would have predicted an SSVEP of 80 Hz. However, in case of entrainment, synchronization of the EEG to the external driving force can happen in multiple Arnold *tongues*. Synchronization typically not only occurs at the frequency of the intrinsic brain oscillator (1:1 Arnold tongue) but also harmonics (*N* ^*^ intrinsic frequency) and subharmonics (intrinsic frequency/*N*) where *N* is an integer (e.g., 1:2 and 2:1). Thus, the 80 Hz flickering LED was entraining the EEG in an 8:1 manner resulting in a 10 Hz oscillation. Harmonic entrainment has been shown on a behavioral level by de Graaf et al. (2013), who found a cyclic pattern of visual detection at a 5.3 Hz entrainment as well as at 10.6 Hz, but not outside the Arnold tongue.

The reported finding of the Arnold tongue with intermittent phase locking at the border of the triangular pattern supports the assumption, that visual entrainment is a valid tool to entrain brain oscillations. Moreover, it reveals evidence for behavioral changes as a consequence of rhythmic visual stimulation to reflect causality. Furthermore, it encourages further investigation using electric and magnetic stimulation, as it confirms that oscillations can generally be entrained by an external driving force.

## Conclusion

We showed for the first time in a systematic analysis that visual flickering stimulation results in entrainment of brain oscillations. Our data demonstrate that the visual cortex responds in a non-linear fashion when being exposed to rhythmic visual stimulation, which contradicts the hypothesis of linear superposition of ERPs. Multiple studies apply a flickering light source to investigate the causal link of brain oscillations and perception (Hanslmayr et al., 2005; Lakatos et al., 2008; Busch et al., 2009; Schroeder and Lakatos, 2009; Jensen and Mazaheri, 2010; Mathewson et al., 2011). By showing that visual rhythmic stimulation modifies ongoing brain oscillations, this study reveals evidence that visual entrainment is capable of probing causality. The effect, however, depends on the frequency's distance from the intrinsic frequency (IAF) as well as on the stimulation intensity, which should both be considered for the design of future experiments. The pattern of the Arnold tongue, as well as intermitted phase locking during rhythmic visual stimulation, reveals strong evidence for entrainment as the underlying mechanism of SSVEPs.

## Author contributions

CH, JK, and AN designed research; CH and AN performed research; CH and AN analyzed data; CH, JK, and AN wrote the paper.

## Funding

The project was supported by the PhD program “Signals and Cognition” and the Special Priority Programme 1665 of the German Research Foundation (DFG grant HE3353/8-1).

### Conflict of interest statement

The authors declare that the research was conducted in the absence of any commercial or financial relationships that could be construed as a potential conflict of interest.
